# Feasibility and validity of Ecological Momentary Assessment in patients with acute coronary syndrome

**DOI:** 10.1186/s12872-020-01774-w

**Published:** 2020-11-27

**Authors:** François A. M. Jean, Igor Sibon, Mathilde Husky, Thierry Couffinhal, Joel Swendsen

**Affiliations:** 1 Department of Psychiatry, Dr Jean Eric Techer Hospital, Calais, France; 2 Centre hospitalier universitaire de Bordeaux, Bordeaux, France; 3 Université de Bordeaux, Bordeaux, France; 4 Institut national de la sante et de la recherche medicale (U1034), Bordeaux, France; 5 Centre national pour la recherche scientifique (INCIA, CNRS 5287), 146 rue Léo Saignat, Bordeaux, France; 6 Ecole Pratiques de Hautes Etudes, PSL University, Paris, France

**Keywords:** Acute coronary syndrome, Ecological Momentary Assessment, Validity, Feasibility

## Abstract

**Background:**

In recent years, Ecological Momentary Assessment (EMA) has expanded rapidly in healthcare research but its application specifically to the field of cardiology has been limited. This study presents essential information concerning the feasibility and validity of EMA in patients with acute coronary syndrome.

**Methods:**

Four months after a first-ever acute coronary syndrome, 47 patients completed EMA five times a day for seven consecutive days concerning their current activities, mood and perceived negativity or positivity of daily events.

**Results:**

Compliance with the repeated electronic assessments was high, and no evidence was found for time-dependent biases such as fatigue or practice effects. The resulting EMA data were found to have high internal validity, high reliability when considering average scores, and low reliability when considering within-person variance.

**Conclusions:**

We found evidence for the feasibility and intrinsic validity of EMA in patients with acute coronary syndrome. Research examining daily life experiences, symptoms and therapeutic adherence in this population can be reinforced through the use of mobile technologies.

## Background

Ambulatory monitoring techniques such as Ecological Momentary Assessment (EMA) have been validated in the investigation of a wide range of diseases and disorders [1–5] as well as in samples ranging from young adolescents to the elderly [6–8]. The contributions of this approach include its capacity to study phenomena in ecologically-valid contexts of daily life, and its ability to collect real-time data overcomes retrospective memory biases associated with traditional and non-electronic research instruments [9, 10]. Importantly, EMA can also be used to provide real-time therapeutic interventions including medication reminders and specific exercises at the moments that they are most needed by patients [11, 12].

It is well-documented that behavioral and psychological variables play a role in the onset as well as recovery from heart disease [13–15], and such variables can have an important impact on the degree to which patients engage in rehabilitation [16]. Ambulatory monitoring of these variables provides an important source of information for both research and clinical intervention. EMA has previously been used in community samples to monitor risk for cardiovascular disease [17], mobile health interventions following cardiac events [18, 19], and non-computerized versions of EMA have been used to examine how mood patterns affect coronary artery calcification [20]. However, despite extensive research examining EMA methodology in other health domains, information is currently lacking concerning the basic metrics of feasibility and validity in cardiology. The reliance of EMA on repeated daily assessments for periods ranging from days to weeks may raise questions relative to its acceptability among certain patients, as well as concerning the typical compliance rate with the multiple daily assessments. In particular, fatigue effects defined as the decline in performance on a prolonged or demanding task [21] have received little attention to date in EMA applications for cardiology, and it is further unknown if practice effects (improved performance due to repeated assessments) or reactive effects (changes in the frequency or intensity of variables as a function repeated assessment) may constitute important biases in this population. In addition to the question of feasibility and biases, information concerning the reliability and validity of data acquired by EMA is essential for encouraging the use of this approach for research purposes and it is a prerequisite to advancing applications for real-time interventions.

In patients with acute coronary syndrome (ACS), the current study uses EMA over a one-week period to examine daily life experiences and activities. The specific objectives are to: (1) estimate initial study acceptance rates as well as compliance with EMA’s multiple daily assessments; (2) examine potential biases and reactive effects associated with the EMA methodology on mood, behavioral and environmental variables; and (3) investigate the split-half reliability adapted to EMA data, as well as internal validity among EMA variables by examining their patterns of association.

## Methods

### Participants

Participants in the present investigation were drawn from a larger study in the cardiology service of a teaching hospital in Bordeaux, Southwest France, that included both an MRI examination and neuropsychological assessments. Participants in the parent study were recruited four months after being admitted for a first-ever acute coronary syndrome. Inclusions were made between September 2009 and December 2012. Persons were excluded if they were younger than 30 years of age, had contraindications for MRI, or if they had a history of neurological disease, major depression, aphasia, or visual, motor or intellectual deficiency preventing the use of the monitoring device. This investigation was approved by the national ethics board and all patients provided written consent to participate. The study protocol conforms to the ethical guidelines of the 1975 Declaration of Helsinki.

### Procedure

Four months after their initial hospitalization, eligible participants were invited to attend a 10-min training session on computerized EMA including the use of a personal digital assistant (PDA) programmed using a modified version of the Purdue Momentary Assessment Tool version 2.1.2. The research assistant ensured that participants understood each question and response choices displayed on the PDA screen during the ambulatory monitoring week. Following this, participants were given a PDA to carry with them for a one-week period. Each PDA was programmed to administer 5 electronic interviews per day. The signals (an auditory alarm) occurred within each of the following time periods: 9:00 am to 11:00 am; 11:00 am to 2:00 pm; 2:00 pm to 5:00 pm; 5:00 pm to 8:00 pm; and 8:00 pm to 10:00 pm. Assessment times were fixed for each participant, and randomized across participants.

### Measures

At each EMA assessment, participants were asked to describe their current location (being at home, friend’s home, partner’s home, relative’s home, medical institution, work, garden, shop, restaurant, administration, other place inside, other place outside), their social company (being alone, being with a friend, partner, relative, colleague, unknown people, pet, or other company), and their activity (work, sport, television, listening to music, in-person conversation, phone or internet conversation, sports or physical leisure, non-physiscal leisure, hygiene, eating, waiting, resting, cooking, shopping, no activity, or other activity). Response options were provided to participants based on items validated in previous EMA investigations [4] and shown to lack reactive effects when questions are presented in the same order at each assessment. Participant were also asked at each assessment to describe their current degree of happy mood, sad mood, anxious mood, anhedonia and perceived negativity or positivity of daily events using a 7-point scale ranging from − 3 ('extremely negative') to 3 ('positive'), in addition to other questions (not analyzed in the current study). Verbatim wording of the full 17-question set and response options are available in supplementary material, Additional file 1: Table S1. The questions were administered in the same order at each assessment and were designed to be completed in 2 min on average. In order to minimize the extent to which responses were influenced by retrospective recall biases, analyses in all samples were limited to assessments that were completed within 20 min of the pre-programmed PDA signal.

### Statistical analyses

We estimated compliance rates as the average number of EMA assessments completed out of all administered assessments. Fatigue effects, practice effects, and reactivity were examined by assessing the frequency, intensity or change in variables (outcomes) a function of study duration (predictor variable). In order to study fatigue effects, we created a dichotomous outcome variable to indicate missing data (coded as ‘1’, compared to completed assessments coded as ‘0’). Practice effects were examined relative to the time needed to complete each EMA assessment in milliseconds (outcome variable). In order to examine reactivity effects, three representative categories were selected based on their relevance and sufficient frequency of occurrence (present in 5% or more of all responses) separately for each domain of activity, environmental context and social contact. For this, we decomposed multinomial variables into individual binary outcome variables, coding ‘1’ for the presence of the category and ‘0’ for all other responses. To assess reliability, we calculated the averages for happy mood, sad mood, anxiety, anhedonia and perceived negativity/positivity of events for each participant and separately for even and odd days, and then computed a permutation pearson correlation to examine their associations. We also calculated the within-person reliability by focal reliability measures (Rc; [22, 23]), with 95% confident intervals and bootstrapping using 10,000 iterations. Internal validity was evaluated through the examination of patterns of associations that would be expected among similar or opposing emotions (e.g. sad and anxious mood were expected to be positively correlated; sad and happy were expected to be negatively correlated). EMA data were analyzed using multilevel models with Hierarchical Linear and Nonlinear Modeling Version 6.08 [24]. Means-as-outcomes (gaussian) models were used for continuous outcomes and Bernoulli (binomial logistic) models for dichotomous outcomes. We used two levels for all models (repeated EMA observations at level 1 being nested within individuals at level 2) with robust standard errors and a random effect of participants to correct dependence due to repeated measures. Happy mood, sad mood, anxious mood, anhedonia and perceived negativity/positivity of events were group-mean centered when used as independent variables (i.e. centered around the individual’s own mean).

## Results

### Participants

Clinical characteristics of the sample and EMA variables are presented in Table 1. The sample was composed of 47 individuals with a mean age of 54.1 years (SD = 7.38), and 19.1% were women. Regarding the nature and treatment of ACS, 59.6% were found to have an ST segment elevation on ECG and 87.2% were treated with coronary stenting. Participants experienced happy mood at a greater average intensity (M = 4.4, SD = 1.05) than either sad (M = 1.9, SD = 1.43) or anxious mood (M = 1.87, SD = 1.39), while anhedonia and perceived negativity/positivity of events were experienced at moderate levels (M = 3.91, SD = 1.53, and M = 3.71, SD = 1.29, respectively). Concerning behavioral variables, participants were most frequently at home (71.3%), with family (42.2%), and watching television (22.4%).

**Table 1:** Description of sociodemographic, clinical and EMA data for the sample

	M	N	SD	%
Age	54.1		7.38	
Sex: female		9		19.1
Troponin (ng/mL)	34.0		48.1	
ST segment elevation		28		59.6
Coronary stenting		41		87.2
Coronary bypass		2		4.3
EMA				
Happy mood	4.40		1.05	
Sad mood	1.90		1.43	
Anxious mood	1.87		1.39	
Anhedonia	3.91		1.53	
Perceived stress	3.73		1.29	
Location				
At home		799		71.3
Being outside		59		5.3
At relative's home		59		5.3
Social interaction				
Alone		330		29.5
With family		475		42.2
With friends		65		5.8
Activity				
Watching television		250		22.4
Talking		64		5.8
Eating		154		13.8

### Feasibility of EMA

While approximately half (45.4%) of eligible participants agreed to take part in the parent study that included MRI, and all of these individuals also agreed to participate in the EMA assessments. No mobile devices were lost or damaged during the study. Overall compliance with the numerous EMA assessments was high, with 79.3% (M = 27.76, SD = 4.16) of the 35 programmed assessments being completed throughout the week, and and 80.9% of participants responded to more than 50% of assessments.

### Biases associated with EMA duration

As demonstrated by Table 2, no fatigue effects were observed in that missing data did not increase as a function of study duration (γ = 0.102; t = 0.969; *p* > 0.05). Practice effects were also absent as participants did not respond more rapidly to daily life assessments as the study progressed (γ = − 13,902.557; t = − 1.744; *p* > 0.05). Concerning reactivity (alterations in variable intensity or frequency as a function of repeated assessments), no effects were observed for happy mood (γ = 0.116; t = 1.576; *p* > 0.05); sad mood (γ = − 0.118; t = − 1.061; *p* > 0.05); anxious mood (γ = − 0.139; t = − 1.223; *p* > 0.05) or anhedonia (γ = 0.013; t = 0.004; *p* > 0.05). However, the perceived negativity/positivity of events changed as the study progressed (γ = − 0.209; t = − 2.161; *p* < 0.05). Concerning reactivity, no significant change was observed as a function of repeated assessments with regard to activity (talking: γ = − 0.173; t = − 1.086; *p* > 0.05; eating: γ = 0.160; t = 1.740; *p* > 0.05; watching television: γ = − 0.080; t = − 0.680), social company (being alone: γ = − 0.117; t = 1.134; *p* > 0.05; being with friends: γ = − 0.059; t = − 0.737; *p* > 0.05; being with family: γ = − 0.138; t = − 1.570; *p* > 0.05), or for environmental contexts (being at home: γ = − 0.002; t = − 0.011; *p* > 0.05; being outside: γ = 0.214; t = 1.455; *p* > 0.05; being at relative’s home: γ = − 0.015; t = − 0.128; *p* > 0.05).

**Table 2:** Fatigue, practice, and reactive effects as a function of day in the study

	γ	SE	T ratio
Fatigue effects	0.102	0.105	0.969
Practice effects	− 13,902.557	7971.901	− 1.744
Reactive effects			
Location			
At home	− 0.002	0.147	− 0.011
Being outside	0.214	0.147	1.455
At relative's home	− 0.015	0.120	− 0.128
Social interaction			
Alone	0.117	0.103	1.134
With family	− 0.138	0.088	− 1.570
With friends	− 0.059	0.080	− 0.737
Activity			
Watching television	− 0.080	0.117	− 0.680
Talking	− 0.173	0.158	− 1.086
Eating	0.160	0.092	1.740
Mood states			
Happy mood	0.116	0.074	1.576
Sad mood	− 0.118	0.111	− 1.061
Anxious mood	− 0.139	0.114	− 1.223
Anhedonia	0.013	0.004	0.140
Perceived stress	− 0.209	0.097	− 2.161*

*p < 0.05. Fatigue = number of missing observation by day of study; Practice = milliseconds needed to complete EMA by day of study; Reactivity = frequency or intensity of variables by day of study

### Reliability and internal validity

In order to assess reliability, all continuous EMA variables were averaged for each participant across even and odd days. These scores for each participant were strongly correlated for happy mood (r = 0.79, t = 8.7, *p* < 0.001), sad mood (r = 0.95, t = 19.8, *p* < 0.001), anxiety (r = 0.83, t = 9.9, *p* < 0.001), anhedonia (r = 0.78, t = 8.3, *p* < 0.001) and perceived negativity/positivity of events (r = 0.87, t = 11.9, *p* < 0.001). The patterns of association among ambulatory monitoring variables are presented in Table 3. As expected, happy mood was negatively associated with sad mood (γ = − 0.333; t = − 4.723; *p* < 0.001) and, conversely, increases in anxiety were associated with greater sad mood (γ = 0.377; t = 7.869; *p* < 0.001). No association was observed between perceived negativity/positivity of events with either anxious (γ = 0.104; t = 1.957; *p* > 0.05) or sad mood (γ = 0.0260; t = 0.495; *p* > 0.05). The focal reliability coefficients were low for all emotions: happy Rc = 0.18 (0.11 0.25), sad Rc = 0.33 (0.26–0.40), anhedonia Rc = 0.07 (0.01–0.14), anxiety Rc = 0.29 (0.22–0.36), perceived negativity/positivity Rc = 0.18 (0.11–0.25).

**Table 3:** Relationships among EMA variables (internal validity)

	γ	SE	T ratio
Mood states			
Sad—happy	− 0.333	0.071	− 4.723***
Anhedonia—happy	− 0.605	0.058	− 10.378***
Anhedonia—sad	0.143	0.059	2.435*
Anxiety and mood			
Anxiety—happy	− 0.253	0.057	− 4.420***
Anxiety—sad	0.377	0.048	7.869***
Anxiety—anhedonia	0.095	0.037	2.578*
Perceived stress and emotions			
Perceived stress—happy	− 0.396	0.067	− 5.924***
Perceived stress—sad	0.0260	0.053	0.495
Perceived stress—anhedonia	0.222	0.040	5.579***
Perceived stress—anxiety	0.104	0.053	1.957

*p < 0.05; ***p < 0.001

## Discussion

Despite increasing interest in Ecological Momentary Assessment in the field of cardiovascular disease, information concerning its basic feasibility and validity has been lacking. The present findings provide strong support for the feasibility and validity of this methodology in ACS patients.

Before the concepts of reliability or validity can be examined, it is first necessary that EMA be acceptable to patients, measurable both in terms of initial study acceptance as well as compliance with the repeated daily assessments. In the current sample, all eligible participants agreed to participate. It is notable that this study was part of a broader investigation involving MRI, and therefore it was not possible to examine the initial participation acceptance rate independently from the other methodologies involved. However, a high overall compliance rate with the repeated daily assessments was also high (79.3%). This rate is similar to what has been observed in healthy samples and in diverse psychiatric populations [4]. Together, these elements provide fundamental support for establishing the feasibility of this methodology for individuals discharged after cardiac events.

The present study showed no fatigue effects, a finding similar to what has been demonstrated in a variety of clinical populations [3, 4]. In addition, no practice effects were found, suggesting that the time it takes participants to complete each assessment did not vary as a function of study duration. Finally, while mood levels were not altered throughout the assessment period, perceived event negativity/positivity changed significantly with study duration. This finding differs from what has been observed with psychiatric samples [3], but may be explained by the fact that the initiation of the study occurred at the time of a scheduled clinical evaluation and it is possible that event perceptions change when patients returned home and to their regular daily routines. However, should reactivity to the repeated daily assessments be observed in future investigations, researchers may wish to control for day of study when conducting analyses of EMA data.

In light of findings supporting the feasibility of EMA and the general absence of time-dependent biases, a final issue concerns the reliability and validity of the resulting data. There are numerous forms of reliability and validity and therefore final recommendations should be based on multiple studies and methods. This particular study examined reliability first by calculating correlations for a given variable assessed across alternating days, and assessed internal validity by examining the presence of correlations in expected directions among parallel or opposed mood states. Strong reliability was observed for all variables examined, and the results also confirm the internal validity of EMA data through the expected correlations among daily life variables, as has been reported in other samples using this technique [4]. However, using the Rc approach that examined within-person variability, low reliability was found for all emotions and the perceived negativity/positivity of events. These findings underscore the fact that these variables are highly fluctuating phenomena, and although their average levels may be reliable, methods such as EMA are necessary for detecting such momentary fluctuations.

These findings should be interpreted in light of several characteristics of the sample and its methodology. First, the moderate sample size may not have been sufficient to detect small but clinically significant effects. While the higher prevalence of acute coronary syndrome in men is also reflected in the current sample, replicating these findings in samples including a larger percentage of women would permit the examination of potential sex differences. In comparison with other studies [25], there was a high proportion of ST elevation in our sample. This may be explained by the fact that very few hospitals with equipment to perform coronary angiography exist in the area where the study was conducted, and therefore patients with ST elevation were referred to this teaching hospital at greater proportions than in other areas. It is also notable that perceived event negativity or positivity was not significantly associated with negative mood states, a finding that may be due to the bimodal scale used for this variable that was different from the unidirectional scales used for mood states. Finally, it should be noted that this investigation did not examine concurrent validity between EMA and traditional self-report assessments commonly used in cardiology. Again for this reason, final conclusions regarding reliability and validity should be based on a diversity of studies using different methods.

## Conclusions

ACS patients demonstrated highly-satisfactory compliance with the repeated assessments provided by EMA, supporting the global feasibility of this methodology in this population. The data generated from this technique were also psychometrically sound, with evidence for reliability as well as internal validity and a general absence of time-dependent biases. A reality in the field of cardiology is that the vast majority of progress made by patients depends on their behavior when they are not accompanied by a clinician. It is indeed during their daily lives that they must remember to take their medications, to avoid risk factors and to practice prescribed exercises. In this way, the present findings strongly suggest that EMA can be used not only for research purposes but also in conjunction with standard treatment to improve medication adherence or to deliver psychosocial interventions. These latter applications are a growing subject of interest, and the lifestyle changes needed after an acute coronary syndrome may therefore be of particular importance for future EMA studies.

## Supplementary information

**Additional file 1**. Original French-language EMA questions and response items

## Data Availability

The datasets generated and/or analyzed during the current study are not publicly available due French regulations for health care research, but are available from the corresponding author upon reasonable individual requests.

## Abbreviations

ACS: Acute Coronary Syndrome
EMA: Ecological Momentary Assessment
PDA: Personal Digital Assistant
