# Supplementary material for: Feasibility and validity of Ecological Momentary Assessment in patients with acute coronary syndrome
Source: BMC Cardiovasc Disord. 2020 Nov 27;20:499. doi: 10.1186/s12872-020-01774-w (PMC7694267; doi:10.1186/s12872-020-01774-w)
Supplement: Supplementary file 1 — Additional file 1. Original French-language EMA questions and response items [file 12872_2020_1774_MOESM1_ESM.docx]

**Supplementary Material**

**Supplementary Material Table S1: Ecological Momentary Assessment questions and response options**

| Question | Type of question | Response options |
| --- | --- | --- |
| Where are you right now? | SAMC | your own home \| partner’s home \| family’s home \| friend’s home \| at work \| restaurant, café or bar \| store or business \| hospital or clinic \| administrative building \| park or garden \| other place inside \| other place outside |
| Who is with you right now? | MAMC | no one (you are alone) \| family \| friends \| partner \| colleagues \| strangers \| a pet \| other |
| What are you doing right now? | MAMC | nothing \| waiting \| napping or resting \| eating \| cooking \| household chores \| work \| shopping \| personal hygiene \| watching tv \| listening to music \| conversation (in person) \| conversation (phone or internet) \| sports or physical leisure \| non-physical leisure \| travel or commuting \| other |
| How happy do you feel right now? | SAMC on a 7-point Likert scale | Not at all happy 1 \| 2 \|3 \| 4 \| 5 \| 6 \| Extremely happy 7 |
| How sad do you feel right now? | SAMC on a 7-point Likert scale | Not at all sad 1 \| 2 \| 3 \| 4 \| 5 \| 6 \| Extremely sad 7 |
| How much interest or pleasure in your activities are you experiencing right now? | SAMC on a 7-point Likert scale | No interest or pleasure 1 \| 2 \| 3 \| 4 \| 5 \| 6 \| Extreme interest or pleasure 7 |
| How anxious do you feel right now? | SAMC on a 7-point Likert scale | Not at all anxious 1 \| 2 \| 3 \| 4 \| 5 \| 6 \| Extremely anxious 7 |
| How tired versus energetic do you feel right now? | SAMC on a 7-point Likert scale | Extremely tired 1 \| 2 \| 3 \| 4 \| 5 \| 6 \| Extremely energetic 7 |
| To what degree are you focused and concentrated right now? | SAMC on a 7-point Likert scale | Extremely focused 1 \| 2 \| 3 \| 4 \| 5 \| 6 \| Extremely distracted 7 |
| To what degree are you feeling physical pain right now? | SAMC on a 10-point Likert scale | No pain at all 1 \| 2 \| 3 \| 4 \| 5 \| 6 \| 7 \| 8 \| 9 \| extreme physical pain 10 |
| When you think of the future, how do you think you will feel? | SAMC on a 7-point Likert scale | Less happy than now 1 \| 2 \| 3 \| no change 4 \| 5 \| 6 \| More happy than now 7 |
| Since the last signal, please note all substances you have used. | MAMC | More medications than prescribed \| alcohol \| cigarettes \| other drug \| no substance |
| Think of what event affected you the most since the last signal and rate its negative or positive impact on you. | SAMC on a 7-point Likert scale | Extremely negative -3 \| -2 \| -1 \| no impact 0 \| 1 \| 2 \| Extremely positive 3 |
| Have you thought of pleasant things since the last signal or things that make you feel good? | SAMC on a 7-point Likert scale | Not at all 1 \| 2 very rarely \| 3 somewhat \| 4 frequently \| 5 Very frequently |
| Have you thought of unpleasant things since the last signal or things that make you feel bad? | SAMC on a 7-point Likert scale | Not at all 1 \| 2 very rarely \| 3 somewhat \| 4 frequently \| 5 Very frequently |
| How many hours did you sleep last night? | SAMC; asked only at first assessment of morning | 1 \| 2 \| 3 \| 4 \| 5 \| 6 \| 7 \| 8 \| 9 \| 10 \| 11 \| 12 \| 13 \| 14 or more hours |
| Have you experienced any of these sleep problems last night? | MAMC; asked only at first assessment of morning | Difficulties falling asleep \| awakening during the night \| awakening early \| difficulties waking up \| other sleep problem \| no sleep problem |
| SAMC : single-answer multiple-choice question ; MAMC : multiple-answer multiple-choice question | | |
